# Mechanical force regulates root resorption in rats through RANKL and OPG

**DOI:** 10.1186/s12903-022-02327-7

**Published:** 2022-07-16

**Authors:** Jianli Zhou, Lijia Guo, Yanji Yang, Yi Liu, Chen Zhang

**Affiliations:** 1 Department of Endodontics, School of Stomatology, Capital Medical University, Tian Tan Xi Li No.4, Beijing, 100050 People’s Republic of China; 2 Immunology Research Center for Oral and Systemic Health, Beijing Friendship Hospital, Capital Medical University, Beijing, People’s Republic of China; 3 Department of Orthodontics, School of Stomatology, Capital Medical University, Beijing, People’s Republic of China; 4 Laboratory of Tissue Regeneration and Immunology and Department of Periodontics, Beijing Key Laboratory of Tooth Regeneration and Function Reconstruction, School of Stomatology, Capital Medical University, Tian Tan Xi Li No. 4, Beijing, 100050 People’s Republic of China

**Keywords:** Orthodontic force, Root resorption, Dental pulp stem cell, Periodontal ligament stem cell

## Abstract

**Background:**

External root resorption is one of common complications of orthodontic treatment, while internal root resorption is rarely observed, and the difference between pulp and periodontal tissues during orthodontic treatment is still unknown. The purpose of this study was to evaluate the effects of orthodontic forces on histological and cellular changes of the dental pulp and periodontal tissues.

**Methods:**

Orthodontic tooth movement model was established in Forty-eight adult male Wistar rats. The distance of orthodontic tooth movement was quantitatively analyzed. The histological changes of pulp and periodontal tissues were performed by hematoxylin–eosin staining, tartrate-resistant acid phosphate staining was used to analyze the changes of osteoclast number, immunohistochemistry analysis and reverse transcription polymerase chain reaction were used to examine the receptor of nuclear factor-κB ligand (RANKL) and osteoprotegerin (OPG) expression. The width of tertiary dentine was quantitatively analyzed. Tartrate-resistant acid phosphate staining and the erosion area of osteo assay surface plate was used to evaluate osteoclast activity.

**Results:**

The orthodontic tooth movement distance increased in a force dependent manner, and reached the peak value when orthodontic force is 60 g. Heavy orthodontic force increased the RANKL expression of periodontal ligament srem cells (PDLSCs) which further activated osteoclasts and resulted in external root resorption, while the RANKL expression of dental pulp stem cells (DPSCs) was relatively low to activate osteoclasts and result in internal root resorption, and the dental pulp tend to form tertiary dentine under orthodontic force stimulation.

**Conclusions:**

Heavy orthodontic forces activated osteoclasts and triggered external root resorption by upregulating RANKL expression in rat periodontal tissues, while there was no significant change of RANKL expression in dental pulp tissue under heavy orthodontic forces, which prevented osteoclast activation and internal root resorption.

## Background

External root resorption is one of the most common complications of orthodontic treatments and previous studies have shown that the incidence of external root resorption during orthodontic treatments is as high as 96% [1]. Factors such as the magnitude of orthodontic force are directly related to the extent of external root resorption [2, 3]. Further, a finite element study confirmed that there is no significant difference between the force distribution of dental pulp and periodontal tissues [4], but heavy orthodontic forces rarely induce internal root resorption [5–7], which indicates that a special defense mechanism exists in dental pulp tissue during orthodontic processes.

Root resorption is initiated by osteoclasts (OCs) [8], which are large, multi-nucleated cells that originate from monocyte/macrophage lineage hematopoietic precursor cells [9]. Under stimulation by macrophage colony-stimulating factor (M-CSF) and receptor activator of nuclear factor kB ligand (RANKL), the mononuclear precursors start to fuse and RANKL can further promote the differentiation of osteoclast precursor cells by binding to the RANK receptor on the surface of osteoclast precursor cells [10, 11]. Osteoprotegerin (OPG) can inhibit the differentiation of OCs by competitive binding to RANKL [11]. One previous study reported an association between the RANKL/OPG ratio and external root resorption [12], indicating that the RANKL/OPG ratio correlates with the root resorption process.

The RANKL/OPG ratio is regulated by many types of cells [13–15]. One earlier study [16] compared the osteoclast-inductive capacities of periodontal ligament stem cells (PDLSCs) from primary teeth and from permanent teeth and reported that PDLSCs from primary teeth promote osteoclast differentiation via the upregulation of RANKL and the downregulation of OPG, which leads to the enhancement of external root resorption, demonstrating that PDLSCs can regulate the physiological root resorption process. However, dental pulp stem cells (DPSCs) in pulp tissue may play a protective role during internal root resorption. One histological study reported that the presence of DPSCs can inhibit the occurrence of internal root resorption, but when DPSCs were replaced by bone marrow stem cells, internal root resorption began [17], which indicates that DPSCs can inhibit internal root resorption but the regulatory mechanism involved is still not clear.

The expression of RANKL/OPG in PDLSCs and in DPSCs is affected by mechanical stimulation [18–20]. Previous research results have shown that after compressive force stimulation, the ratio of RANKL/OPG in PDLSCs from patients with aggressive external apical root resorption was significantly upregulated [12], but whether heavy orthodontic forces regulate internal root resorption through RANKL/OPG expression in DPSCs is still unknown. Thus, we hyposis that there was no RANNKL expression in pulp tissues under heavy orthodontic force, and we used HE staining, TRAP staining, immunohistochemical staining and hard tissue milling slides for in vivo studies, and RT-PCR, TRAP stainig, osteo assay surface plate for in vitro studies, aiming to explore the effects of heavy orthodontic forces on dental pulp and periodontal tissues with in vivo and in vitro studies, to provide a basis to characterize the mechanism of root resorption.

## Methods

**In vivo experiments:** HE staining, TRAP staining, immunohistochemical staining, hard tissue milling slides were performed for observation of pulp and periodontal tissue changes.

### Animals

This study conformed to ARRIVE (Animal Research: Reporting of In Vivo Experiments) guidelines and the animal study protocol was approved by the Animal Ethics Committee of the Capital Medical University School of Stomatology. Forty-eight adult male Wistar rats, aged 6 weeks and each weighing 220 g, were used in this study. All animals were provided by SPF Biotechnology Co., Ltd. (Beijing). Before the experiment, the rats were housed in a normal experimental environment for 1 week to acclimatize to that environment.

### Orthodontic tooth movement model

The rats were simple randomized into four groups according to the magnitude of orthodontic force applied (Control, 40 g Group, 60 g Group, 100 g Group, twelve rats in each group). After general anesthesia (0.4% phenobarbital sodium, 10 ml/kg), half of the rats in each group were treated with a tetracycline marker (25 mg/kg) to allow observation of tertiary dentine changes, after which the orthodontic devices were set according to a previous study [21]. Briefly, a nickel-titanium coiled spring (wire size 0.25 mm; diameter 0.012 inch; Tomy International, Inc., Japan) was placed between the incisor and the left maxillary first molar of each rat, after which a flowable restorative resin (3 M ESPE, MN, USA) was used to fix the spring with the force applied of 0, 40, 60 or 100 g. All the rats survived before their execution, they were sacrificed on day 14 after inhalation anesthesia (5% isoflurane for induction and 23% for maintenance).The confounders in the experiment were not explicitly controlled.

### Measurement of tooth movement distance

The distance from the distal marginal ridge of the left maxillary first molar to the mesial marginal ridge of the left maxillary second molar was measured as the tooth moving distance using a stereo microscope (SWZ 1000, Tokyo, Japan). All samples were sent to three independent observers for measurement in a blinded fashion.

### Histological analysis

The maxillae from rats that hadn’t received a tetracycline injection were perfused in 4% paraformaldehyde (PFA) for 24 h, and were then decalcified in 10% EDTA (pH 8.0). After decalcification, all samples were embedded in paraffin and cut into 5 μm thick sections for further staining. Slides of each group were randomly selected for hematoxylin–eosin (H&E) staining. Soft tissue and hard tissue responses of dental pulp and periodontal tissue responses were observed using a light microscope (Media Cybernatics, USA).

### Tartrate-resistant acid phosphate assay

Tartrate-resistant acid phosphate (TRAP) staining was performed in accordance with a previous study [22], after which TRAP-positive cells in the dental pulp and on the pressure side of periodontal tissues of the left maxillary first molar were counted. The results are reported as numbers of OCs in dental pulp and in periodontal tissues.

### Immunohistochemistry

Tissue sections were deparaffinized in xylene, then dehydrated in alcohol and incubated in citric acid solution (MXB Biotechnologies, Fuzhou, China) for 10 min at 90 °C to remove surface antigens. After rinsing with PBS, Cell and Tissue Staining Kits (R&D Systems, MN, USA) were used to detect antigens according to the manufacturer's protocols. Tissue sections were stained overnight at 4 °C with an anti-RANKL antibody (1:100; ab45039, Abcam, Cambridge, UK) or an anti-OPG antibody (1:200; ab9986, Abcam, Cambridge, UK), and after rinsing with PBS, they were incubated for 1 h with a secondary antibody (1:2000; ab97240, Abcam, Cambridge, UK; 1:1000 ab6721, Abcam, Cambridge, UK), a fluorescence microscope system (OLYMPUS, Tokyo, Japan) was used to capture images.

### Analysis of hard tissue milling slides

The maxillae from rats that had received a tetracycline injection were dissected, cleaned and fixed, then embedded in resin glue and sectioned at 50–70 microns with an EXAKT cutting machine (EXAKT 310 CP, Germany), a fluorescence microscope system (OLYMPUS, Tokyo, Japan)) was used to capture images.

**In vitro experiments:** RT-PCR, TRAP staining and osteo assay surface plate were used for cellular changes after compressive force stimulation.

### Culture of human DPSCs and PDLSCs

This study was approved by the Ethics Committee of the School of Stomatology, Capital Medical University (CMUSH-IRB0KJ0PJ-2018-03) and informed consent were obtained form all donors. Teeth were obtained from three human donors aged 13–20 years during the extraction of impacted third molars in the Maxillo-Facial Department of the Capital Medical University School of Stomatology. DPSCs and PDLSCs were cultured according to a previously published method [23]. Briefly, cell suspensions of DPSCs and PDLSCs were cultured in 10 cm dishes in DMEM/F12 (1:1) containing 10% fetal bovine serum (FBS), 100 U/ml penicillin (PS) and 100 μg/ml streptomycin. All cells were cultured in a humidified atmosphere with 5% CO_2_ at 37℃ and the culture medium was changed every 3 days. After culture and expansion, the third to fifth passages of DPSCs and PDLSCs were used for the following experiments.

### Application of mechanical compressive forces

DPSCs and PDLSCs were seeded separately in 6-well plates at 2.5 × 10^4^. After the cells reached 80% confluence, a 40 g compressive force was exerted on the 6-well plates for 12 h according to a previous study [24], with cells without force stimulation used as controls. After the application of force, the cells were processed for analyses of differentiation, and osteoclast-inducing medium was prepared using DPSC/PDLSC culture medium containing 30 ng/ml recombinant mouse M-CSF (rmGM-CSF; R&D Systems, MN, USA) and 100 ng/ml RANKL (R&D Systems, MN, USA).

### Total RNA extraction and RT-PCR

Total RNAs were extracted from DPSCs and from PDLSCs after the application of mechanical force using TRIzol reagent (Invitrogen, CA, USA) according to the manufacturer’s protocol, after which cDNAs were synthesized using a reverse transcript kit (Takara Biotechnology, Japan). Real-time PCR was performed using a SYBR Green PCR kit (Takara Biotechnology, Japan) and the sequences of RNA are as follows: GAPDH (5ʹ-CCAAGGAGTAAGACCCCTGG-3ʹ, 5ʹ -AGGGGAGATTCAGTGTGGTG-3ʹ), RANKL (5ʹ-ATCGTTGGATCACAGCACATCAG-3ʹ, 5ʹ-GGATGTCGGTGGCATTAATAGTGAG-3ʹ) and OPG (5ʹ-GCACCGTCAAGGCTGAGAAC-3ʹ, 5ʹ-TGGTGAAGACGCCAGTGGA-3ʹ).

### OC induction and TRAP staining

Mononuclear cells were isolated from 6-week-old C57 mice according to a previously published method [25]. The cells in suspension were then seeded in 24-well plates and were cultured with differentiating medium harvested from DPSCs and PDLSCs after mechanical compressive force stimulation; cells cultured with α-MEM containing 10% FBS, 1% PS, 30 ng/ml M-CSF and 100 ng/ml RANKL served as a positive control. After 5 days, TRAP staining was performed using a leukocyte phosphatase kit (Sigma-Aldrich, USA) and cells with 3 or more nuclei are considered OCs. TRAP-positive cells in 5 randomly selected areas in each plate were counted using a light microscope (OLYMPUS, Tokyo, Japan).

### OC induction and activity on osteo assay surface plates

Mononuclear cells were isolated from 6-week-old C57 mice as detailed above and were seeded on osteo-assay surface plates. The osteoclast-inducing medium was harvested from DPSCs and from PDLSCs after mechanical compressive force stimulation; cells cultured with α-MEM containing 10% FBS, 1% PS, 30 ng/ml M-CSF (R&D Systems, MN, USA) and 100 ng/ml RANKL (R&D Systems, MN, USA) served as a positive control. After 5 days, the erosion areas in 5 randomly selected areas in each plate were measured using a light microscope.

### Statistical analysis

Power analysis using NCSS PASS 2000 (Statistical Software, UT, USA) indicated that 12 rats per group would be needed to provide statistical power (1–β) of 0.8 and a type I error rate of 0.05. SPSS 20.0 software was used for statistical analysis. Independent sample t-test was used to examine the difference between two groups. One-way analysis and Tukey’s test was performed for the comparison of multiple groups. Kruskal–Wallis tests were used for samples with heteroscedasticity. *P* values < 0.05 are considered statistically significant differences.

## Results

### A 60 g orthodontic force is ideal for orthodontic tooth movement.

**Fig. 1:**
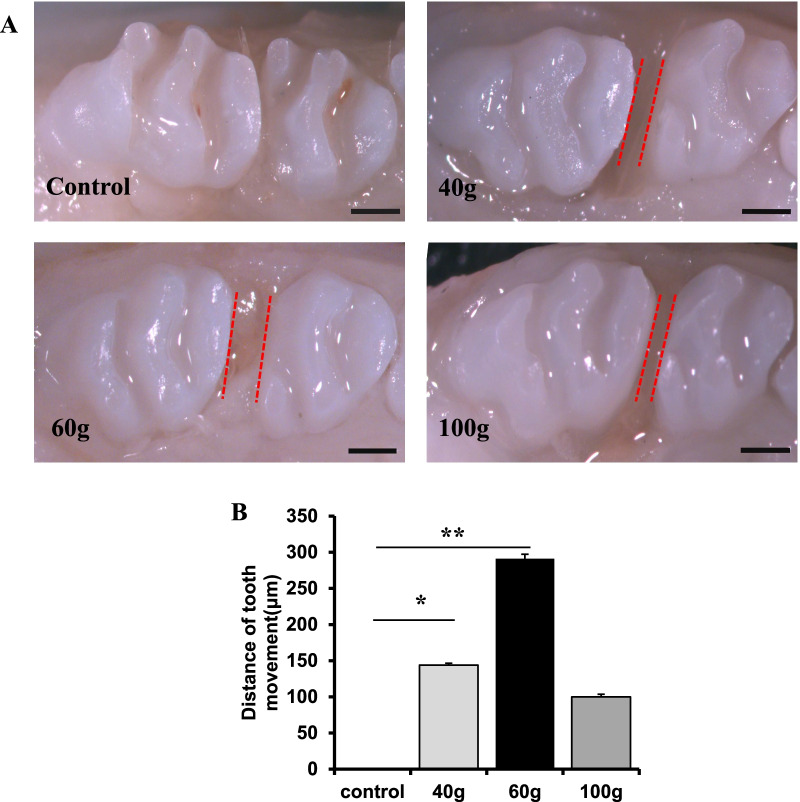
A 60 g orthodontic force is optimal to induce tooth movement. **A**, **B** The distance from the distal ridge of the first molar to the mesial ridge of the second molar is considered the tooth movement distance. The movement distance of the first molar increased in a force-dependent manner and reached a peak at 60 g (*P* < 0.01). When the orthodontic force was greater than 60 g, the tooth movement distance decreased. Red dotted lines indicate the tooth movement borders. All results are representative of three repeated experiments. *p < 0.05; **p < 0.01

The movement distance of the first molar increased in a force-dependent manner and reached a peak at 60 g (Fig. 1B, *P* < 0.01), but when the orthodontic force was greater than 60 g, the tooth movement distance decreased (Fig. 1A, B).

### A heavy orthodontic force induces external root resorption.

**Fig. 2:**
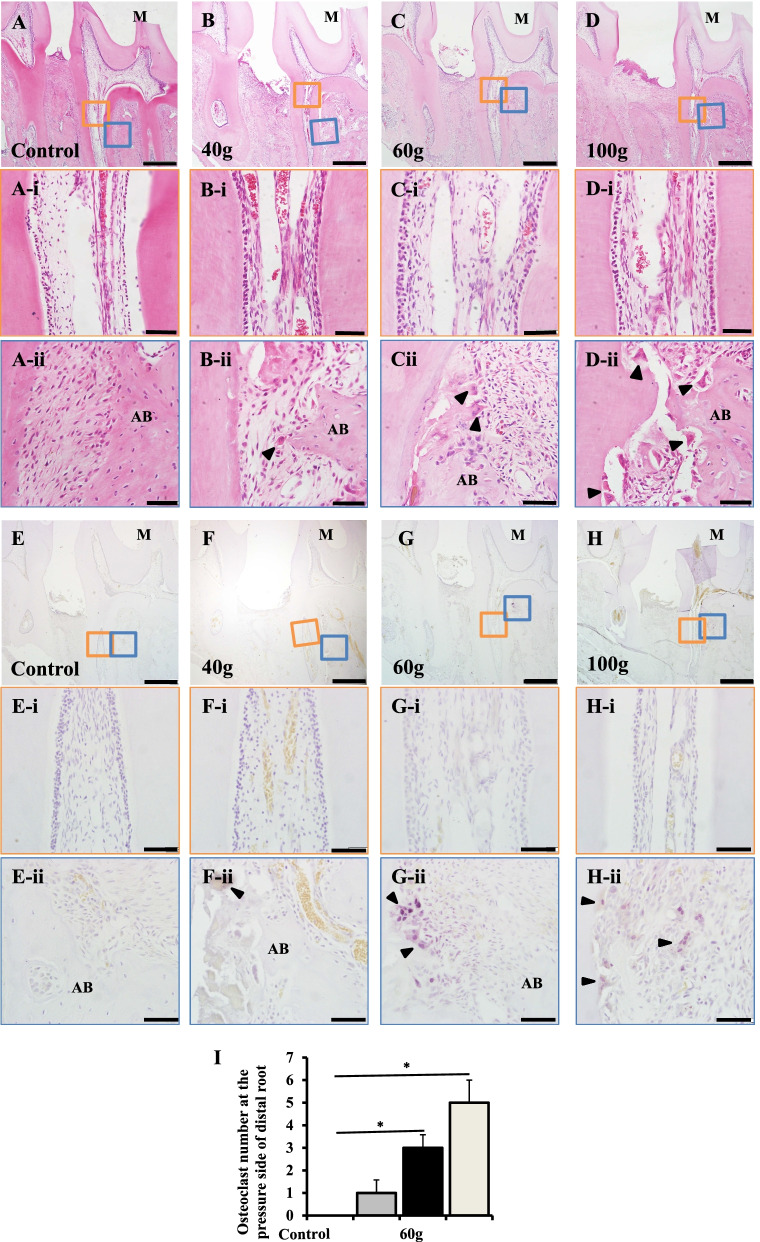
A heavy orthodontic force induces external root resorption. **A**–**D** Images of HE staining show the effects of different orthodontic forces on dental pulp and periodontal tissues assessed by light microscopy. The orange-colored rectangles indicate areas shown at higher magnification in (**A-i**–**D-i**). No inflammation cells or multinucleated cells were observed in coronal pulp tissues of the control or experimental groups. The blue-colored rectangles indicate areas shown at higher magnificent in (**A-ii**–**D-ii**). Multi-nucleated osteoclasts (OCs) were observed in the heavy force group (100 g) at the mesial side of the distal root of the left maxillary first molar. M: mesial, D: distal. **E**–**H** Light microscope images of TRAP staining showing the effects of different orthodontic forces on the expression of TRAP-positive cells in dental pulp and periodontal tissues after orthodontic treatment. The results show that the number of OCs on the pressure side of the distal root of the left maxillary first molar increased following the increase of orthodontic forces. The orange-colored rectangles indicate areas shown at higher magnificent in (**E-i**–**H-i**). No OCs were observed in dental pulp tissues of the control or experimental groups. The blue-colored rectangles indicate areas shown at higher magnificent in (**E-ii**–**H-ii**). **I** The number of OCs increased significantly in the heavy force group (100 g) compared to the control group (*P* < 0.05). M: mesial, D: distal, T: tooth, AB: alveolar bone, arrowheads indicate OCs. All results are representative of three repeated experiments. *p < 0.05. The scale bars in (**A**–**H**) are 100 μm, and are 50 μm in (**A-i**–**H-i**), and in (**A-ii**–**H-ii**)

Histological changes of dental pulp and periodontal tissues were observed using a light microscope. In the control group (orthodontic force = 0 g), no multi-nucleated OCs or resorption lacunae were observed in pulp and periodontal tissues (Fig. 2A). In the 40 g and 60 g groups, a few OCs were observed in the alveolar bone on the mesial side of the distal root of the left maxillary first molar (Fig. 2B, C). In the 100 g group, several resorption lacunae were identified in the alveolar bone and root surface on the mesial side of the distal root of the left maxillary first molar (Fig. 2D). TRAP staining showed that the number of TRAP-positive cells in the 60 g and 100 g groups was significantly higher than in the control group (*P* < 0.05) (Fig. 2E–I).

### A heavy orthodontic force promotes the expression of RANKL in periodontal tissues.

**Fig. 3:**
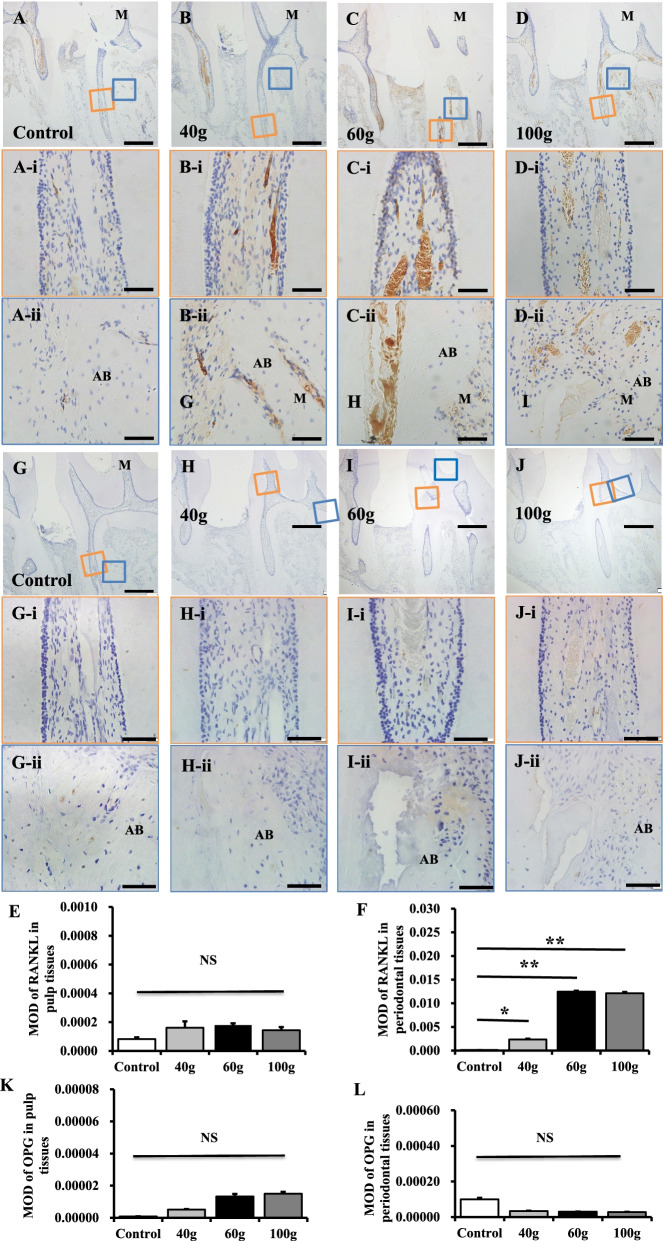
A heavy orthodontic force promotes the expression of RANKL in periodontal tissues, but does not affect OPG expression in dental pulp or periodontal tissues. **A**–**D** To further evaluate the expression of RANKL in dental pulp and periodontal tissues, we performed immunohistochemical staining. The results show that orthodontic forces promote the expression of RANKL on the compressive side of periodontal tissues. The orange-colored rectangles indicate areas shown at higher magnification in (**A-i**–**D-i**). **E** Semi-quantitative analysis of the percentage of RANKL-positive cells in pulp tissues, the RANKL expression was very low in pulp tissues, there was no significant difference among groups. The blue-colored rectangles indicate areas shown at higher magnification in (**A-ii**–**D-ii**). **F** Semi-quantitative analysis of the percentage of RANKL-positive cells in periodontal tissues on the pressure side of the first molar. The percentage of RANKL-positive cells on the compressive side of periodontal tissues in the experimental groups increased in a force-dependent manner (*P* < 0.01). **G**–**J** Immunohistochemical staining showing that there is no significant difference in the expression of OPG in dental pulp and periodontal tissues. The orange-colored rectangles indicate areas shown at higher magnification in (**G-i**–**J-i**). The blue-colored rectangles indicate areas shown at higher magnification in (**G-ii**–**J-ii**). **K**, **L** Semi-quantitative analysis of the percentage of OPG-positive cells in periodontal tissues on the pressure side of the first molar. The percentage of OPG-positive cells in dental pulp (**K**) and periodontal tissues (**L**) did not change significantly (*P* > 0.05). M: mesial, D: distal, T: tooth, AB: alveola bone. All results are representative of three repeated experiments. *p < 0.05; **p < 0.01. The scale bars in (**A**–**J**) are 100 μm, while those in (**A-i**–**J-i**, **A-ii**–**J-ii**) are 50 μm

The RANKL/OPG ratio is one of the key factors for OC activity. To examine whether a heavy orthodontic force influences RANKL/OPG expression in dental pulp and/or periodontal tissues, we performed immunohistochemical staining. RANKL expression in periodontal tissues increased significantly after orthodontic force stimulation (Fig. 3A–F, *P* < 0.01), while OPG expression in pulp and periodontal tissues was very low and there was no significant difference between the experimental groups and the control group (Fig. 3G–L, *P* > 0.05).

### An orthodontic force promotes the formation of tertiary dentine.

**Fig. 4:**
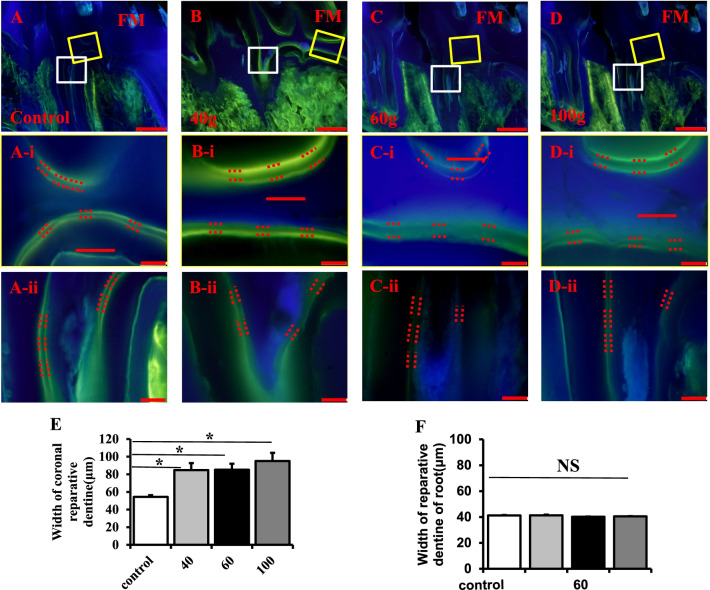
A heavy orthodontic force promotes the formation of tertiary dentine. **A**–**D** Hard tissue milling slides show that the width of the tertiary dentine of the coronal pulp increased after orthodontic tooth movement. The yellow-colored rectangles indicate areas shown at higher magnification in (**A-i**–**D-i**), the white-colored rectangles indicate areas shown at higher magnification in (**A-ii**–**D-ii**). **E** Quantitative analysis showing that the width of tertiary dentine of the coronal pulp increases in a force-dependent manner, the heavy force group (100 g) exhibits a wider tertiary dentine compared with the control group (*P* < 0.05), **F** while no significant change was observed for the width of tertiary dentine of the root (*P* > 0.05). M: mesial, D: distal, P: pulp; D: dentine. All results are representative of three repeated experiments. *p < 0.05. The scale bars in (**A**–**D**) are 100 μm, while those in (**A-i**–**D-i**, **A-ii**–**D-ii**) are 50 μm

Since tertiary dentine has protective effects on the dentine beneath it, we measured the width of tertiary dentine after orthodontic treatment. Hard tissue milling slides showed that the width of the tertiary dentine of the coronal dental pulp increased significantly after orthodontic treatment (Fig. 4A–E, *P* < 0.05) while no significant change was observed for the width of tertiary dentine of the root (Fig. 4F, *P* > 0.05).

### A mechanical compressive force promotes OC differentiation through the upregulation of RANKL expression in PDLSCs.

**Fig. 5:**
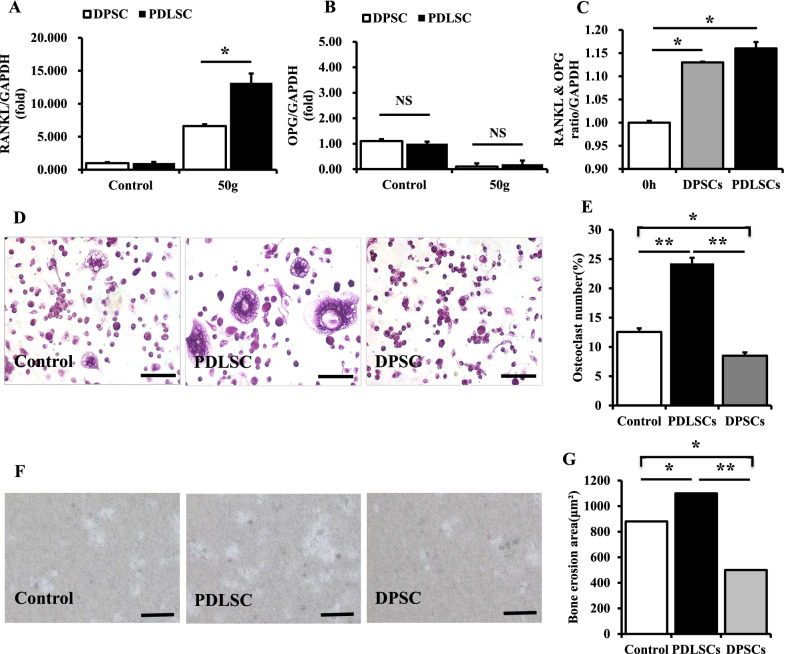
A mechanical compressive force upregulates RANKL expression by PDLSCs, which further promotes osteoclast differentiation of mononuclear cells. **A**–**B** RT-PCR results showing that mechanical compressive forces significantly up-regulate RANKL expression by PDLSCs and it was significantly higher than DPSCs (*P* < 0.05), while the OPG expression of PDLSCs and DPSCs showed no significant difference (*P* > 0.05). **C**–**D** TRAP staining shows that the cell culture medium of PDLSCs after compressive stimulation can promote the osteoclast differentiation of mononuclear cells. The TRAP positive cell number was significantly higher than in the positive control group and in the DPSC group (*P* < 0.05), and OCs in the PDLSC group created significantly more erosion areas on osteo assay surface plates (*P* < 0.05). All results are representative of three repeated experiments. *p < 0.05; **p < 0.01. The scale bars in (**C**) and (**E**) are 10 μm

The immunohistochemical staining results showed that the secretion of RANKL by PDLSCs treatment with a 50 g force was significantly higher than by DPSCs (Fig. 5A, *P* < 0.05), while there was no significant difference in OPG expression between PDLSCs and DPSCs (Fig. 5B, *P* > 0.05). And the RANKL/OPG ratio was significantly higher in DPSCs and PDLSCs (Fig. 5C, *P* < 0.05). TRAP staining showed that the number of OCs in the PDLSC group was significantly higher than in the positive control group and in the DPSC group (Fig. 5D–E, *P* < 0.01), while the number of OCs in the DPSC group was significantly lower than the positive control group (Fig. 5D–E, *P* < 0.05). Further, the erosion area in the PDLSC group was significantly higher than in the positive control group and the DPSC group (Fig. 5F–G, *P* < 0.01), but the erosion area in the DPSC was significantly lower than the positive control group (Fig. 5F–G, *P* < 0.05).

## Discussion

This study was designed to evaluate the effects of orthodontic forces on histological changes of dental pulp and periodontal tissues, and the effects of DPSCs and PDLSCs on osteoclast activation under mechanical compressive stress. The results of this study do not support the null hypothesis that there is no RANKL expression within the pulp tissue, but its low expression levels are insufficient to activate odontoclasts and initiate internal root resorption. We observed that a heavy orthodontic force induced external root resorption by upregulating RANKL expression in periodontal tissues, but no internal root resorption was observed. Further, the RANKL/OPG expression in dental pulp tissues was not significantly changed and orthodontic force promoted the formation of tertiary dentine. To evaluate the role of the RANKL/OPG axis in the root resorption process, we applied mechanical compressive forces on DPSCs and on PDLSCs. The results showed that heavy compressive forces can promote the expression of RANKL in PDLSCs, which further promotes the differentiation of OCs from mononuclear cells and creates larger erosion areas on osteo assay surface plates.

No internal root resorption was observed in any of the four groups. Hard tissue milling slides showed that the thickness of tertiary dentine of the coronal pulp of the first molar increased in a force-dependent trend. Tertiary dentine [26] is the reactive production of pulp tissue under stimulation, the formation of which can protect the pulp tissue and maintain its vitality. Pulpal responses and the formation of tertiary dentine are complex biological processes. A previous in vitro study confirmed that mechanical stress can affect the differentiation of DPSCs [27]. One study [28] found that a mechanical compressive force can promote the secretion of BMP2 by DPSCs, while another study [29] confirmed that the secretion of DSPP and DMP1 by DPSCs is upregulated under mechanical tensile stress, which suggests that mechanical stress stimulation can promote the odontoblastic differentiation of DPSCs [30]. Further, a study [31] of the predentine thickness of the coronal pulp of rats under an orthodontic force showed that the width of predentine in the coronal pulp increased after orthodontic treatment. The thickness of predentine reflects active dentine formation [32, 33], which proves that dentine formation and the reparative process in coronal pulp tissue is active during orthodontic treatment, but the regulatory mechanism still needs further study.

Orthodontic root resorption is related to many factors. The magnitude of the orthodontic force applied is one of the decisive factors for root resorption [34]. Previous studies have found that a higher orthodontic force is related to greater root resorption, and a heavy orthodontic force will lead to external root resorption compared with a light orthodontic force [34, 35]. OCs were observed on the pressure side of the root in the heavy force group [12]. Our results also demonstrate that the number of OCs on the pressure side of the maxillary first molar increased in a force-dependent manner, and when the orthodontic force was 100 g, external root resorption occurred, and OCs were observed on the mesial root surface of the distal root of the maxillary first molar, which is in accordance with an earlier study [12].

The RANKL/RANK/OPG axis is the classic regulator for external root resorption, which regulates the activation and differentiation of OCs. The balance of the RANKL/OPG system may be affected during orthodontic tooth movement [11, 19]. Studies have shown that on the pressure side of orthodontically treated teeth, the expression of RANKL is greater under a heavy force than under the optimum force [12, 36] and OCs induced by RANKL further lead to root resorption. Our immunohistochemical staining also proves that the RANKL/OPG ratio on the pressure side of the left maxillary first molar increased with increasing orthodontic force.

Collectively, the in vivo results demonstrated that the RANKL/OPG ratio in pulp tissue was not significantly changed by heavy orthodontic force, thus no internal root resorption was observed. And we also found that orthodontic force could stimulate the dentine formation of pulp tissues, but the underlying regulation mechanism needed further study. The results also proved that the RANKL/OPG ratio on the pressure side of periodontal tissue was significantly increased by heavy orthodontic force, which induced external root resorption on the pressure side of the root.

The RANKL/OPG ratio directly reflects the balance of osteogenesis and osteoclastogenesis [11, 19]. Previous research has demonstrated that mechanical forces can regulate the differentiation of DPSCs and PDLSCs, which plays an important role in root resorption [37]. The mechanical stress induced by orthodontic treatment can stimulate PDLSCs directly and can transmit through enamel, dentin, and thus further stimulate inner pulp tissues. Therefore, we evaluated the effects of the mechanical microenvironment on the differentiation of DPSCs and PDLSCs in vitro. Our results demonstrate that a heavy mechanical compression promotes the expression of RANKL in DPSCs and PDLSCs, and the expression of RANKL in PDLSCs is significantly higher than in DPSCs. The RANKL/OPG ratio was significantly increased both in DPSCs and in PDLSCs. Although the RANKL expression of DPSCs increased after mechanical compression, the RANKL secreted by DPSCs under compressive force stimulation was still relatively lower than positive control, thus TRAP positive cells number in DPSC group was significantly lower than positive control group, and the erosion areas in DPSC group were significantly lower than positive control group as well. While the RANKL secreted by PDLSCs was higher than other groups, so we observed more TRAP positive cells in PDLSC group, and the activated OCs created larger erosion areas on osteo assay surface plates. The in vitro results demonstrated that a heavy mechanical compression promoted the expression of RANKL in PDLSCs which initiated the OCs activation, while the RANKL expression of DPSCs was insufficient to activate the OCs.

Collectively, a heavy orthodontic force increases the RANKL/OPG ratio in periodontal tissues which further activates OCs and results in external root resorption. Further, the expression of RANKL in dental pulp tissue is not sufficient to activate OCs and the formation of tertiary dentin protects the dentin from internal root resorption.

Although our in vivo and in vitro data suggest that RANKL/OPG played an important role in internal and external root resorption process, many cells and cytokines were also involved in this process, more studies should be performed in the future.

## Conclusions

A heavy orthodontic force results in external root resorption by activating OCs through increased RANKL expression in PDLSCs, while RANKL expression in DPSCs is relatively low and is not sufficient to activate OCs for internal root resorption.

## Data Availability

All data generated or analysed during this study are included in this published article.

## Abbreviations

RANKL: Receptor of nuclear factor-κB ligand
OPG: Osteoprotegerin
PDLSCs: Periodontal ligament srem cells
DPSCs: Dental pulp stem cells
OCs: Osteoclasts
M-CSF: Macrophage colony-stimulating factor
PFA: Paraformaldehyde
TRAP: Tartrate-resistant acid phosphate
BMP2: Bone morphogenetic protein 2
DSPP: Dentin phosphoprotein
DMP1: Dentin matrix acidic phosphoprotein 1
